# Multi-tissue transcriptomics of a unique monozygotic discordant twin case of severe progressive osseous heteroplasia

**DOI:** 10.1016/j.gendis.2023.05.001

**Published:** 2023-06-19

**Authors:** Alberto Gómez-Carballa, María José Currás-Tuala, Sara Pischedda, Miriam Cebey-López, José Gómez-Rial, Irene Rivero-Calle, Jacobo Pardo-Seco, Xabier Bello, Sandra Viz-Lasheras, Antonio Justicia-Grande, Julián Montoto-Louzao, Alba Camino-Mera, Isabel Ferreirós-Vidal, Máximo Fraga, José R. Antúnez, Rodolfo Gómez, Federico Martinón-Torres, Antonio Salas

**Affiliations:** a Genetics, Vaccines and Infections Research Group (GENVIP), Instituto de Investigación Sanitaria de Santiago, Santiago de Compostela, Galicia 15706, Spain; b Translational Pediatrics and Infectious Diseases, Department of Pediatrics, Hospital Clínico Universitario de Santiago de Compostela, Servicio Galego de Saúde (SERGAS), Galicia 15706, Spain; c Laboratorio de Inmunología, Servicio de Análisis Clínicos, Hospital Clínico Universitario de Santiago de Compostela, Servicio Galego de Saúde (SERGAS), Galicia 15706, Spain; d Unidade de Xenética, Instituto de Ciencias Forenses (INCIFOR), Facultade de Medicina, Universidade de Santiago de Compostela, and GenPoB Research Group, Instituto de Investigación Sanitaria, Hospital Clínico Universitario de Santiago de Compostela, Servicio Galego de Saúde (SERGAS), Galicia 15706, Spain; e Musculoskeletal Pathology Group, Instituto de Investigación Sanitaria de Santiago, Servicio Galego de Saúde (SERGAS), Santiago de Compostela, Galicia 15706, Spain; f Department of Pathology, Hospital Clínico Universitario de Santiago de Compostela (SERGAS), Galicia 15706, Spain; g Unidad Integrada de Biobancos, Biomodelos 3D y Modelos Animales, IDIS (Instituto de Investigación Sanitaria de Santiago de Compostela), Galicia 15706, Spain; h Centro de Investigación Biomédica en Red de Enfermedades Respiratorias (CIBER-ES), Madrid, Spain

Progressive osseous heteroplasia (POH) is an ultra-rare autosomal dominant disabling disorder characterized by heterotopic ossification (HO). It is caused by heterozygous inactivating mutations in the *GNAS* (guanine nucleotide-binding protein alpha-stimulating activity polypeptide) gene. However, the molecular mechanisms underlying HO remain poorly understood. As a treatment for POH is not yet available, the identification of the mechanisms driving POH in affected tissues using gene expression may be of great help to underestand the molecular basis of POH and develop new therapeutic approaches.

We collected samples from a unique case of two monochorionic twin sisters diagnosed with POH, both carrying the same inactivating heterozygotic mutation in *GNAS* (*565-568delGACT*) but with very different clinical manifestations. One sister showed an aggressive and disabling phenotype, while the other was virtually asymptomatic. The molecular micro-environment of affected tissues may play a crucial role in subcutaneous HO and the different progression of the disease. We analyzed gene expression patterns in “healthy” skin (from the buttock) and skin attached to a HO (from the knee), as well as HO samples from the scapular and abdominal regions of the most affected patient, and compared them to analogous tissue samples from healthy controls (Table S1 and Fig. S1). The analysis used a gene expression Nanostring panel consisting of over 700 genes related to osseous metabolism. In addition, we compared the whole blood transcriptome (RNA-seq) and miRNAs profile of both twins to find possible systemic signals explaining the differential phenotype.

The results showed that HO samples collected from the abdominal and scapular regions exhibited very similar expression patterns (Fig. 1A). This similarity was reflected in the high correlation between log_2_FC values from both plate samples using controls as reference (*r* = 0.82, *P*-value = 2.2E-16; Fig. 1A and Table S2). However, we observed differential expression patterns in the sample from the skin attached to the HO (knee) and the skin from an apparently “healthy” area (buttock), resulting in a poor correlation between log_2_FC values when compared with controls (*r* = 0.28). The buttock skin showed a gene expression profile similar to the control sample (Fig. 1B and Table S3).

**Figure 1:**
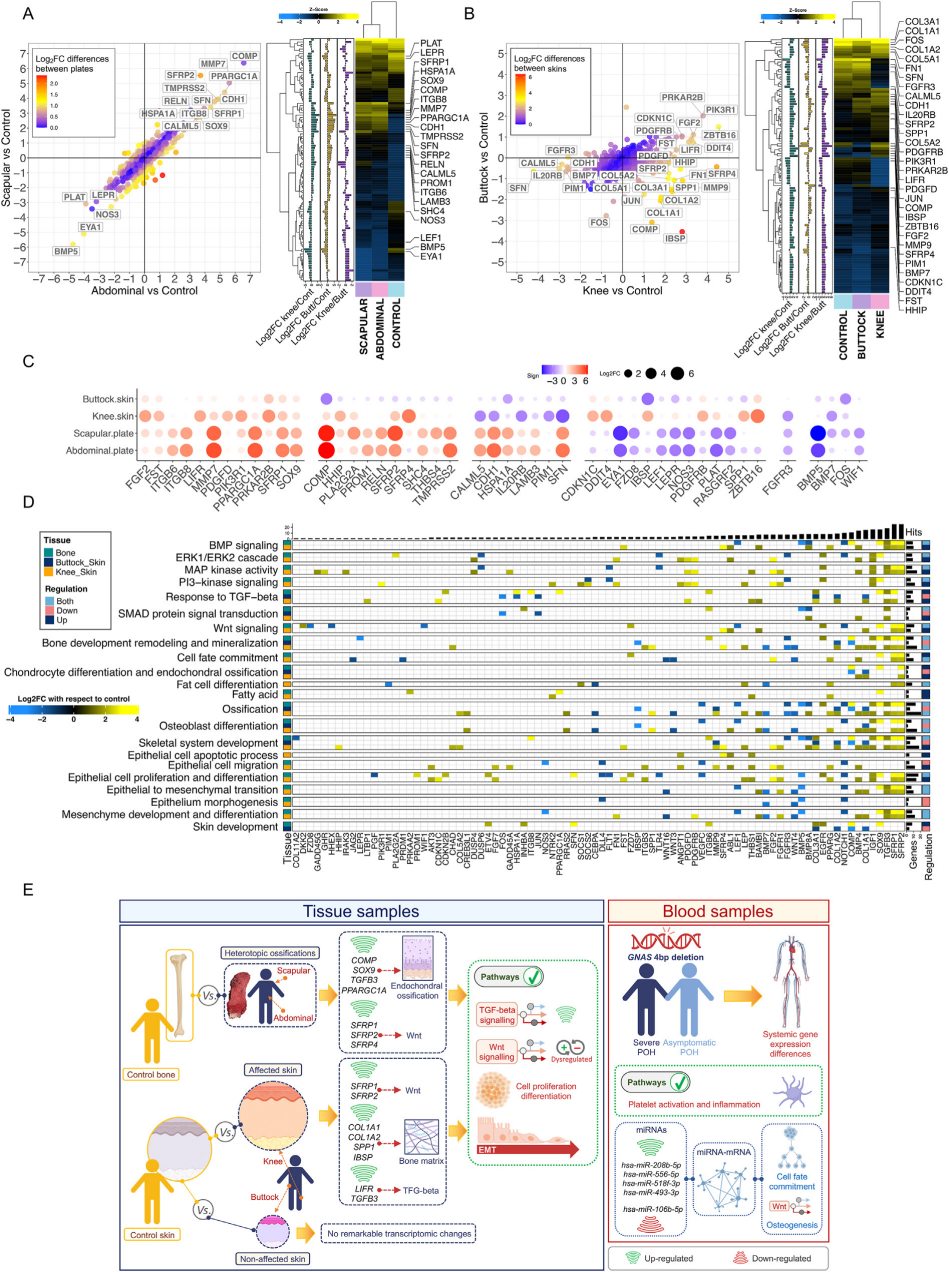
Transcriptomics of progressive osseous heteroplasia. **(A)** Gene expression profile of heterotopic ossification samples. Correlation plot of log_2_FC values obtained from the comparisons between heterotopic ossification samples and control separately. Gene names in the figure correspond to genes with a log_2_FC > |2.5| in any of the comparisons (left). Heatmap and cluster analysis of the gene expression patterns of heterotopic ossification samples as well as control bone. Only genes with a log_2_FC > |1.5| between samples are represented (right). **(B)** Gene expression profile of skin samples. Correlation plot of log_2_FC values obtained from the comparisons between skin samples and control separately. Gene names in the figure correspond to genes with a log_2_FC > |2.5| in any of the comparisons (left). Heatmap and cluster analysis of the gene expression patterns of skin samples from the affected patient as well as control skin. Only genes with a log_2_FC > |1.5| between samples are represented (right). **(C)** Top differentially expressed genes in different tissues. Plot showing most differentially expressed genes with respect to control samples. The size of the bubbles is proportional to the log_2_FC value and the color indicates over or under regulation with respect to control samples. Only genes with a log_2_FC > |2.5| were included (and with >100 counts in any of the samples compared). **(D)** Pathway analysis and genes involved. Heatmap displaying the main biological processes detected in the pathways analysis and the genes involved. The color of the target genes indicates the log_2_FC values of the samples with respect to controls. Only genes with a log_2_FC > |1.5| were included in the analysis. **(E)** Graphical summary. Main findings from the transcriptomic analysis of different tissues analyzed in this study. The figure was built using resources from Biorender (https://biorender.com/).

Histopathological studies indicated that HO in POH is mainly formed through an intramembranous ossification^1^. However, the transcriptomic profiles suggest that both HO samples would have an endochondral origin, as the genes with higher log_2_FC values were closely related to cartilage development and chondrocyte differentiation (*e.g.*, *SOX9*, *COMP*, or *TGFB3*; Fig. 1C; Fig. S3). Pathways related to endochondral bone growth and cartilage development were also over-activated in these samples (*P*-adjusted < 0.01; Fig. 1D and Table S4). *SOX9* plays a main regulatory role in endochondral ossification promoting mesenchymal condensation and the formation of the cartilaginous template. We also found an up-regulation of the *PPARGC1A* gene, which has been found to be over-expressed during chondrogenesis of mesenchymal stem cells. *PPARGC1A* can act as a co-activator for *SOX9* and co-regulate chondrogenesis after stimulation through TGF-β. Co-over-expression of *PPARGC1A* and *SOX9* can activate the expression of other extracellular matrix chondrogenic proteins, such as thrombospondin-5 (encoded by *COMP* gene), which plays a critical role in cartilage bone matrix organization. *COMP* was the most over-expressed gene in the HO samples (log_2_FC > 6) when compared to the control sample (Table S2).

The expression of major bone matrix genes (*COL1A1*, *COL1A2*, *SPP1*, and *IBSP*) were found to be highly expressed in the control bone sample (Table S2) with similar expression values detected for the HO samples, supporting the notion of its osseous nature. However, in the knee skin samples, these genes were all over-expressed compared to the control sample (Table S3), suggesting the establishment of a favorable ossification micro-environment in the subcutaneous region, potentially promoting HO progression (supplementary material).

To maintain an adequate regulation of bone metabolism, G*s*α balances the activities of Wnt and Hh signaling (supplementary material). While the involvement of Wnt signaling in the differentiation of mesenchymal stem cells to chondrocytes and osteoblasts is known, its regulation remains unknown. In this study, the expression levels of intracellular inhibitors of Wnt were similar between controls and patient samples in both HO and skin tissues. However, extracellular antagonists of Wnt were found to be strongly up-regulated in both HO (*SFRP1*, *SFRP2*) and knee skin (*SFRP1*, *SFRP2*, *SFRP4*), with respect to control samples, suggesting a potential drop-off in Wnt signaling (Fig. 1C). It is important to note that *SFRPs* can also act as Wnt agonist and expand the signaling area of Wnt pathway. Additionally, activation of the Wnt pathway can trigger some Wnt inhibitory molecules such as *AXIN2*. Therefore, caution should be exercized when interpreting Wnt-related inferences based on experimental findings (supplementary material).

Interestingly, our data identified TGF-β as the most relevant up-regulated pathway in the HO and knee skin samples when compared to control (Table S4 and Fig. 1D; Fig. S3). TGF-β is known to regulate several cell processes, but also bone formation, remodeling, and repair, and it is required in all phases of chondrogenesis. Notably, the up-regulation of *TGFB3* gene in the HO and the affected skin samples is particularly relevant, since its gene product, TGF-β3, has been shown to induce chondrogenic differentiation in adipose-derived stem cells.^2^ TGF-β also plays an important role in HO-inducing mesenchymal stem cell migration and recruitment, initiating the HO process and promoting angiogenesis. TGF-β inhibitors have been proposed as a treatment against HO.^3^ Furthermore, TGF-β stimulates osteoblast differentiation and the expression of *LIF* (supplementary material). Consequently, the over-activation of TGF-β together with the over-expression of *LIFR* by the cells from the knee skin sample (Log_2_FC = 3.13; Fig. 1C), could establish a hypersensitive local microenvironment that would contribute to a more efficient response to *LIF*. Notabley, TGF-β can also have a pro-oncogenic activity promoting cell stemness, motility, epithelial to mesenchymal transition (EMT), and inhibiting immune cell functions. EMT-related pathways were found significantly associated with POH in the HO and knee skin samples (Table S4 and Fig. 1D) and might be considered as a possible mechanism promoting the appearance, growth, and dispersion of the HO in a way that resembles a metastatic tumor progression. Studies on fibrodysplasia ossificans progressiva (FOP) have highlighted the importance of EMT in the initial stage of HO formation.^4^

The analysis of blood transcriptome in non-affected twin *vs* affected one (Table S5) revealed significantly over-represented categories related, among others (Table S6 and Fig. S6) to inflammation, as well as some significantly depleted pathways involved to osseous metabolism, specifically, Wnt and MAPK pathways. Additionally, a significant over-representation of genes that participate in platelet activation (*PF4*, *GP9*, *TREML1*, and *MYL9*) was found*,* which has been demonstrated to enhance the release of inflammatory factors *in vitro* and boost the differentiation/proliferation of mesenchymal stem cell, fibroblast proliferation, angiogenesis, and extracellular matrix deposition.^5^ Platelet concentrates have been tested in different contexts due to their capacity to improve bone formation and healing.

Finally, among the five top differentially expressed miRNAs between both twins (Table S7), miR-106 b-5p (under-expressed) and miR-493–3p (over-expressed) might be involved in osteogenic regulation and cell fate commitment, respectively (Table S8, 9; supplementary material).

Overall, gene expression profiles suggest an important endochondral component in HO of the severe patient, with strong involvement of the TGF-β pathway in the molecular micro-environment change and HO generation. In addition, a possible contribution of the EMT to the HO appearance and disease progression may be considered. In blood, we found differences between both twins pointing to systemic signals of imbalance in some pathways related to bone formation and homeostasis; as well as two miRNAs that would deserve further attention as possible therapeutic targets (Fig. 1E). This study represents the first gene expression analysis conducted on fresh human tissular and systemic samples in the context of POH disease.

## Ethics declaration

The studies involving human participants were reviewed and approved by the Ethics Committee of Clinical Investigation of Galicia (CEIC ref. 2019/325). Written informed consent to participate in this study was provided by the participants' legal guardian/next of kin. Written informed consent was obtained from the individual(s), and minor(s)' legal guardian/next of kin, for the publication of any potentially identifiable images or data included in this article.

## Author contributions

All authors contributed to the article and approved the submitted version. Specifically, FM-T, MC-L, JP-S, IR-C, MJC-T, AS, JG-R, AG-C, and IF-V conceptualized and designed the study; IR-C, JG-R, and AJ-G carried out clinical characterization and follow-up; SP, SVL, JM-L, AC-M, and AG-C carried out the gene expression assays; MF and JRA carried out the histological analysis, and AG-C, MJC-T, and XB analyzed the data. AG-C, AS, and RG drafted the initial manuscript. All authors reviewed and revised the manuscript and approved the final manuscript as submitted.

## Conflict of interests

The authors declare that there are no competing interests.
