# Supplementary material for: Multi-tissue transcriptomics of a unique monozygotic discordant twin case of severe progressive osseous heteroplasia
Source: Genes Dis. 2023 Jun 19;11(3):100981. doi: 10.1016/j.gendis.2023.05.001 (PMC10808913; doi:10.1016/j.gendis.2023.05.001)

Supplementary Material

Multi-tissue transcriptomics of a unique monozygotic discordant twin case of severe progressive osseous heteroplasia

Alberto Gómez-Carballa, María José Currás-Tuala, Sara Pischedda, Miriam Cebey-López, José Gómez-Rial, Irene Rivero-Calle, Jacobo Pardo-Seco, Xabier Bello, Sandra Viz-Lasheras, Antonio Justicia-Grande, Julián Montoto-Louzao, Alba Camino-Mera, Isabel Ferreirós-Vidal, Máximo Fraga, José R. Antúnez, Rodolfo Gómez, Federico Martinón-Torres, Antonio Salas

Index

1. Additional background

2. Material and Methods

2.1. Samples

2.2. Gene expression analysis of tissue simples

2.3. Gene expression analysis of blood simples

2.4. miRNAs gene target interactions

2.5. Over-representation enrichment analysis

3. Additional Results

3.1. Gene expression patterns in heterotopic ossification and skin samples

3.2. Pathways analysis in heterotopic ossification and skin samples

3.3. Blood mRNA and miRNAs profile in affected and unaffected twins

4. Additional Discussion

4.1. Dysregulation of cell dynamics-related processes

4.2. Endochondral origin of the heterotopic ossifications

4.3. Dysregulation of Wnt pathway

4.4. Local TGF-β involvement

4.5. Systemic signals of severe POH

5. Study limitations

6. References

7. Legend to the supplementary tables

8. Supplementary Figures

1. Additional background

Progressive osseous heteroplasia (POH) is an ultra-rare and disabling disease, characterized by progressive extra-skeletal ossifications of subcutaneous and deep connective tissue, skeletal muscle, and fascia (*1, 2*). It is caused by heterozygous inactivating mutations in the Guanine Nucleotide binding protein Alpha Stimulating (*GNAS*) gene, a complex locus that encodes for the alpha-subunit of heterotrimeric stimulatory G protein s (G*s*α). Although this G*s*α is the major product of this gene, it also generates multiple products through the management of different promoters and initial exons (*3*). Once G*s*α is activated by the union of a ligand to the G protein-coupled receptors (GPRCs), the canonical function of active G*s*α is to trigger adenylyl cyclase increasing cyclic adenosine monophosphate (cAMP) levels, resulting in the activation of different cAMP-regulated proteins, such as protein kinase A (PKA) (*4*). This locus is imprinted, showing allele-specific regulation of transcription, with paternal, maternal o biallelic expression depending on the tissue type (*5*-*7*). POH is mainly produced by mutations affecting the paternal allele of *GNAS* (*8*).

POH can be distinguished from other related disorders also characterized by mutations at the *GNAS* gene e.g. pseudohypoparathyroidism (PHP) type Ia (PHP-Ia), Albright's hereditary osteodystrophy (AHO) and osteoma cutis (OC). Whilst non-POH syndromes are characterized by a superficial bone lesion involving skin and subcutaneous fat, in POH these lesions can progress into deeper tissues (*1, 2, 8*). The absence of endocrine alterations is also distinctive of POH (*9, 10*). In POH and other *GNAS*-related disorders, the ectopic bone is usually formed through an intramembranous process from mesenchymal stem cells. However, in some cases, endochondral or a combination of both endochondral and intramembranous ossification has been observed to some extent (*9*). The skin disorder known as Type 2 segmental mosaicism has been also proposed as a possible explanation for the POH feature since it can arise from different *GNAS* inactivation backgrounds and, moreover, it is not transmitted as a common Mendelian trait (*11*). In other congenital anomalies with monogenetic basis, such as the McCune-Albright syndrome caused by mutations in *GNAS1* gene, mutant cells can only survive as a segmental mosaic, as otherwise they would lead to fetal intrauterine death (*12*). Another condition that

causes heterotopic bone formation is fibrodysplasia ossificans progressive (FOP), but it has a different genetic basis, and the typical lesions are comparable to those produced by non-POH *GNAS*-related syndromes (*13, 14*). FOP is caused by an activating missense mutation in the bone morphogenetic protein type I receptor gene (*ACVR1*) that typically produces an ectopic cartilage formation prior to ossification – a process known as endochondral ossification (*14*).

The molecular mechanisms underlying heterotopic ossification (HO) remain poorly understood. It is well known that cell differentiation processes involved in bone homeostasis and formation are ruled by a precise balance between specific up- and down-regulated gene-sets from several important signaling pathways such us Hedgehog (Hh) (*15-17*), Wnt (*18-20*), Bone Morphogenic Protein (BMP) (*14, 21*), or mTOR (*22, 23*). Studies in animal models showed that a correct balance between Hh and Wnt/b-catenine is key to maintain an appropriate bone homeostasis (*24*). Changes in the expression levels of these pathways might lead to skeletal disorders such as osteoporosis, characterized by a generation of low density bone tissue, or/and ectopic bone formation in soft-tissues (*16*). In POH and similar pathologies, loss of function of the G*s*α subunit leads to an imbalance between both signaling routes producing an up-regulation of basal Hh signalling and other osteogenic signals (*15*); this imbalance provides a favorable micro-environment for the ectopic osteoblast differentiation and bone formation (*16, 24, 25*). Recently, a positive feedback between the axis Yes-Associated Protein (YAP) and Sonic Hedgehog (SHh) caused by loss of *GNAS* function has been proposed as the core mechanism for heterotopic ossification formation (*26*). This YAP-SHh positive loop would trigger osteoblast differentiation in adjacent mesenchymal cells favoring the HO spread. Several pathways have been highlighted as potential therapeutic targets in POH (*27, 28*). In a recent study using adipose-derived stromal progenitor cells in a mouse model of HO lacking *GNAS* function, the importance of tissue micro-environment to generate HO in subcutaneous tissue was highlighted (*25*). Likewise, the impact of *GNAS* inactivation on the adipose tissue structure was observed, represented by a reduction in adipose tissue volume and an increase in extracellular matrix components prior to the formation of the HO.

Ultra-rare diseases represent a great challenge in biomedical research due to the small number of cases reported worldwide, limiting opportunities for research. Particularly for POH, less than 60 affected individuals have been identified worldwide and reported in the literature (*10*). However, determining its real incidence in the general population is hindered by the lack of recognition and/or misdiagnosis of the disease derived from symptoms overlapping with other similar pathologies or the clinical heterogeneity of POH, usually leading to a limited pathophysiology knowledge and the lack of clinical and 'omic' data related to the disease. Moreover, symptoms appear at birth or within the first few weeks of life and, particularly in the most severe cases, they usually progress with age, making early diagnosis and treatment even more difficult. The identification of the mechanisms driving POH phenotype in affected tissues using molecular approaches, such as gene expression, may be of great help to overcome some of the aforementioned challenges hampering the study of rare and ultra-rare diseases. So far, molecular studies on POH have been carried out *in vitro* or in animal models. In our study, we analyze gene expression patterns in samples collected from a unique case of two monochorionic twin sisters born in 2010 and diagnosed with POH when they were 6 months old, both carrying the same *de novo* pathogenic mutation in the *GNAS* locus (heterozygotic deletion of 4 base pairs: *565-568del 565–68delGACT*; Exon 7) but with very different clinical manifestations of the disease. The clinical course of this twin case and pharmacological treatments have been recently described (*29, 30*).

Our study constitutes the first attempt at exploring the gene expression patterns on affected tissues from a severe case of POH.

2. Material and Methods

2.1. Samples

Two skin and two HO samples were collected from the severely affected patient. The samples represented different areas of the body and were obtained following successive palliative surgeries carried out in 2015, 2016 and 2018 (see **Table S1** for details).

One skin sample was extracted from the affected knee in the ossified leg (skin attached to the heterotopic bone). This ossification was described as

intramembranous by the pathologists, fitting the common pattern described in previous reports of POH ossifications (**Fig. S1**). The other skin sample was collected from the buttock and was not attached to the heterotopic bone; it was therefore, considered to represent a 'healthy' tissue sample. Heterotopic bone samples were obtained from the scapular and abdominal regions; the bone plates showed a comparable phenotype (**Fig. S2**). Additionally, we collected skin and bone samples from healthy individuals.

Samples were immersed in RNA*later* solution just after the surgical removal to preserve RNA integrity. RNA from skin samples was isolated using the RNeasy Mini kit (Qiagen) and following the manufacturer recommendations. RNA extraction from bone and ossification samples required an initial treatment using liquid nitrogen. Thus, the samples were placed in liquid nitrogen and ground using a mortar and pestle (prechilled in liquid nitrogen). Once the liquid nitrogen was evaporated, ground tissue was transferred to a 1.5 ml tube (RNAse free), covered with Trizol reagent, and frozen until the RNA isolation was performed. Before RNA isolation, the tubes containing the samples and Trizol reagent were thawed, and supernatant was transferred to a new tube, ensuring that the rest of the tissue remained in the tube. We added 100µL of $CHCl_3$ per 500µL of Trizol to the tube, shook the sample for 3 minutes and left it 5 minutes at room temperature. After this step, RNeasy Mini kit (Qiagen) was used for RNA purification following the manufacturer recommendations.

Blood samples (2.5ml) were collected in PAXgene tubes (BD). RNA was isolated using PAXgene blood miRNA extraction kit (Qiagen) following manufacturer recommendations and carrying out the on-column DNase I treatment during the extraction process.

Quality control of the RNA samples was performed with TapeStation 2200 (Agilent).

2.2. Gene expression analysis of tissue samples

Gene expression patterns of tissue samples were analyzed using the *n*Counter technology from Nanostring (NanoString Technologies; Seattle), a technological platform that allows to study up to 800 mRNAs in a single reaction using limited amounts of RNA as input and in absence of any enzymatic reaction (amplification or cDNA synthesis). We used a mRNA Nanostring commercial panel

(https://nanostring.com/products/ncounter-assays-panels/oncology/ncounter-pancancer-pathways-panel/) containing 770 genes from 13 canonical signalling pathways (including, Cell Cycle, Apoptosis, Hedgehog, Wnt, DNA Damage Control, Transcriptional Regulation, Chromatin Modification, and TGF-β). In addition, this panel includes 40 reference genes for data normalization.

A quality control check of raw data obtained from this mRNA panel was carried out to verify the absence of technical problems. Data from mRNA panel was subjected to a technical normalization using the geometric mean of the positive spike controls and the mean plus 2 standard deviations of the negative spike controls as a conservative value for background thresholding. To correct for differences in sample input we normalized the data by selecting the optimal number and best reference gene candidates from the panel through the GeNorm algorithm (*31*) following the procedure described in our previous study (*32*). We disregarded reference genes with less than 50 counts in any of the samples. We used a different normalization approach for the miRNA panel.

Log_2FC was calculated by subtracting the arithmetic mean of log2 counts of gene A in the patient sample and the arithmetic mean of log_2 counts of gene A in the reference sample (control). Heatmaps were built using *ComplexHeatmap* R package (*33*). Scatterplots of log_2FC values and Pearson correlation indexes were obtained for the tissue comparisons. All graphics were created using *R* software (*34*).

2.3. Gene expression analysis of blood samples

RNA-seq analysis of blood samples was carried out following the standardized method described in (*35*). The quality of the libraries was analyzed using Bioanalyzer 2100 (High Sensitivity assay), and quantification was determined by real-time PCR in LightCycler 480 (Roche). Prior to clusters generation in cbot (Illumina), an equimolar pooling of the libraries was performed. The pool of the cDNA libraries was sequenced using paired-end sequencing (100 bp) on Illumina HiSeq 2500 and a read quality of 85%>Q30. Post-processing of the RNAseq data involved a quality check of the raw data obtained using the *FastaQC* software (http://www.bioinformatics.babraham.ac.uk/projects/fastqc), removal of problematic 3' end and adapters using the *Trimmomatic* package (*36*)and the reads mapping against the human reference genome (GCh38,v.104) using

ultrafast universal RNA-seq aligner *STAR* (*37*). Secondary and low-quality alignments were removed using *SAMtools* (*38*). To count the number of reads mapping to each gene (counts per gene) we used *RSEM* software (*39*).

Given the sample size limitation to carry out a standard differential expression analysis, we followed an exploratory approach through *NOISeq* package (*40*) which can detect differentially expressed genes (DEGs) between two experimental conditions when no biological or technical replicates are available. *NOISeq* employs non-parametric methods and can simulate technical replicates based on the assumption that expression values (counts) follow a multinomial distribution, where the probability for each gene in the multinomial distribution is the probability that a read maps to the gene. These mapping probabilities are approximated using the expression values in the single sample from each of the corresponding experimental conditions. Firstly, we filtered low expressed genes by removing genes with a sum of expression value across all samples below 1 (in counts per million) and eliminated genes with inconsistent expression values using a threshold for the coefficient of variation per condition of 100. Normalization of the expression data was performed using the TMM (trimmed weighted average of the M-values) method. Afterwards, we simulated replicates using a simulation value of 20% of the reads and a variability of 2% as tuning parameters. The simulation was carried out by generating 10 replicates for each condition (asymptomatic HOP *vs*. severe HOP). Fold change differences (M) and absolute expression differences (D) were then used to assess the probability of differential expression (Pr[differential expression] / Pr[non-differential expression]). We considered a probability cut-off point of 0.99 to assess a gene differential expression between both conditions.

The enrichment analysis of the differentially expressed genes was carried out with the *goseq* (*41*) package, which takes into account the length bias of the genes present in the dataset. We used the Wallenius distribution to approximate the true null distribution.

In addition to RNAseq, blood samples were also analyzed using a commercial *n*Counter Nanostring panel (https://nanostring.com/products/ncounter-assays-panels/immunology/mirna/) to explore differences between both twins, this panel targets 827 miRNA of biological relevance. Following Nanostring recommendations, we normalized the

data using the top 100 expressed miRNAs after background noise thresholding using the same procedure as in the mRNA panel.

This panel could not be used for the tissue samples because miRNAs were not isolated for these samples.

2.4. miRNAs gene target interactions

The package *miRNAtap* was employed (*42*) to predict gene targets for key miRNA with higher differential expression between twins. The miRNAs prediction analysis was carried out based on the databases *PicTar*, *DIANA*, *TargetScan*, *MiRanda* and *miRDB*. The geometric mean score was used to rank each gene. For each miRNA, a gene target was selected if appearing in at least 3 out of the 5 databases. Heatmap of top target genes was generated with the *ComplexHeatmap* R package (*33*).

2.5. Over-representation enrichment analysis

We took an over-representation (ORA) approach to identify biological processes related to the differences found in the gene expression assay between patient samples and controls. We used genes with a $log_2FC > |1.5|$ with respect to control samples. The abdominal and scapular samples showed similar expression patterns; in fact, when compared against controls, both samples yielded the same list of genes above a given log_2FC threshold. In agreement, ORA analysis detected the same significant pathways. Therefore, only one of the results was finally shown.

In the case of miRNAs detected in blood samples and to avoid false positive results, we only selected those miRNAs meeting two criteria: *i*) counts values above 100 in any of the samples and *ii*) a $log_2FC >|1.5|$ in the gene expression comparison for the gene-target analysis. Only target genes with aggregate rank values under 10 were used for the downstream enrichment analysis.

We carried out the functional pathways analysis through the *Clusterprofiler* (*43*) R package. We applied the Benjamini-Hochberg procedure for multiple test correction and *P*-value and *Q*-value thresholds were set both to 0.05. We interrogated GO (Gene Ontology) as reference database, and a maximum gene-set size of 550 and a minimum gene-set size of 10. We used the package

enrichplot (*44*) for graphically presentation of the results. Fold enrichment was calculated as the quotient of gene ratio (number of genes of interest annotated to the gene set / total number of genes of interest) and background ratio (size of the gene-set / size of all the unique genes annotated in the reference database).

3. Additional Results

3.1. Gene expression patterns in heterotopic ossification and skin samples

The genes that showed the highest over-expression ($log_2FC > 4$; **Table S2; Fig. S3**) in both ossification samples) were *COMP* (Cartilage Oligomeric Matrix Protein; log_2FC [abdominal] = 6.6; log_2FC [scapular] = 6.4), *MMP7* (Matrix Metallopeptidase 7; log_2FC [abdominal] = of 5.6; log_2FC [scapular] = 5.0) and *PPARGC1A* (log_2FC [abdominal] = 5.3; log_2FC [scapular] = 4.4 in abdominal / scapular). *COMP* and *MMP7* encode for proteins with different functions in the extracellular matrix (ECM), whilst *PPARGC1A* (Peroxisome Proliferator-Activated Receptor Gamma) plays a central role in the regulation of cellular energy in all tissues and regulates both carbohydrate and lipid metabolism. In contrast, *BMP5* (Bone Morphogenetic Protein 5) and *EYA1* (EYA Transcriptional Coactivator and Phosphatase 1) genes displayed the lowest log_2FC in both plate samples ($log_2FC < - 4$), with functions related to bone and cartilage development and DNA repair processes respectively. Genes with the most different expression between abdominal and scapular plates were *FOS*, *MMP9*, *PLA2G2A*, *SFRP2*, *SOCS3* and *THBS4* (**Fig. S4A**). Genes *PLA2G2A SFRP2* and *THBS4* showed down-regulation in controls and up-regulation in the abdominal and more remarkably, the scapular plate, while *MMP9* and *SOCS3* showed an opposite log_2FC expression pattern in scapular and abdominal plates compared to the control sample. Specifically, *SFRP2*, which has a relevant role in the normal bone formation, was found to be related to ectopic ossification processes.

The most important up-regulated genes in the knee skin sample (when compared to control) were the transcription factor *ZBTB16* ($log_2FC = 4.53$), *SFRP4* ($log_2FC = 4.44$), *FGF2* ($log_2FC = 4.44$), *DDIT4* ($log_2FC = 4.44$), *PIK3R1* ($log_2FC = 4.44$) and *LIFR* ($log_2FC = 3.13$), whereas the most down-regulated were *SFN* ($log_2FC = -4.21$) and *IL20RB* ($log_2FC = -3$) genes (**Fig. S3)**.

Expression changes found in the buttock skin sample (*vs*. control) were not as high as those detected in the knee sample, with *COL2A1* (log_2FC = 2.43) and *PRKAR2B* (log_2FC = 2.02) being the most up-regulated genes and, in contrast, *IBSP* (log_2FC = -3.55), *COMP* (log_2FC = - 3.10) and *FOS* (log_2FC = - 2.78) exhibiting the most important under-expression values.

However, genes showing the largest expression differences between both skin samples (when compared to controls) are over-expressed in knee but under-expressed in buttock (**Fig. S4B**): *IBSP*, *MMP9*, *COL1A1*, *COL3A1* and *COL1A2* (**Table S3**). These genes are involved in extracellular matrix organization, tissue remodeling and skin and bone development processes. The highest expression difference was found for the *IBSP* (Integrin Binding Sialoprotein) gene (Log_2FC = 2.8 and -3.6 in knee and buttock, respectively). This gene encodes for a mineralized tissue-specific non-collagenous protein, which constitutes one of the most common proteins in the bone matrix.

A few genes have comparable expression patterns in both skin samples (when compared to the control tissue), including *HSPB1* (Heat Shock Protein; log_2FC= -1.5 in both skin samples), the protein kinase cAMP-dependent *PRKAR2B* (log_2FC= 2.0 and 3.8 in buttock and knee, respectively) and *ZBTB16* (log_2FC= 1.5 in buttock and 4.5 and knee, respectively) (**Fig. 1B**). These genes may be involved in the differentiation of different cell types (*HSPB1*), regulation of balance of adipose tissue (*PRKAR2B*) and in cell cycle progression and development of differentiated tissues (*ZBTB16*). Moreover, a role in bone metabolism has been described for all these genes.

3.2. Pathways analysis in heterotopic ossification and skin samples

A pathway analysis of these two HO samples indicates that the most important over-regulated signal corresponds to the transforming growth factor beta pathway (TGF- β), which comprises a family of cytokines involved in the homeostasis of connective tissue and skeletal system, with a critical role in osteogenesis (*P*-adjusted = 1.88 E-07; **Fig. S5A; Table S4**).

Among other significant terms related to bone metabolism we have detected relevant dysregulated pathways, such as Wnt pathway (over-expression of *DKK2*, *EGFR*, *FZD7*, *SFRP1*, *SFRP2* and *SOX9* genes and under-expression of *NOTCH*, *HHEX*, *LEF1* and *FZD8* genes); bone morphogenetic protein

signalling pathway (up-regulation of *COMP*, *SFRP2*, *SFRP1*, *SOX9*, *TGFB3* and down-regulation of *NOTCH1*, *BMP8A*, *LEF1* and *BMP5*); IP3K signalling (up-regulation of *SOX9*, *IGF1*, *EGFR* and down-regulation of *SOCS2*, *FGFR3*, *FLT1*), MAPK activity (up-regulation of *SFRP2*, *SFRP1*, *IGF1*, *TGFB3*, *EGFR* and down-regulation of *DUSP6*, *FLT1*, *TLR4*) and SMAD proteins signal transduction (over-expression of *ITGB6*, *INHBA* and under-expression of *BMP8A*, *BMP5*). Processes related to skeletal development, biomineralization, ossification and osteoblast differentiation were also found to be altered (up- and down-regulation).

In addition, we identify an imbalance in routes related to epithelial cells, such as proliferation (up-regulation of *CDKN2B*, *EGFR*, *FZD7*, *IGF1*, *SFN*, *SFRP1*, *SFRP2*, *SOX9* and down-regulation of *BMP5*, *DLL4*, *FLT1*, *NOTCH1*, *PGF*, *TLR4* and *VEGFC*) or epithelial to mesenchymal transition (up-regulation of *SFRP2*, *SFRP1*, *SOX9*, *TGFB3* and down of *NOTCH1*, *LEF1*, *BMP5*) was spotted.

We also found an up-regulation of pathways involved in chondrocyte development and differentiation as well as endochondral bone growth and morphogenesis, with *COMP* and *SOX9* being the most representative genes in these processes. Interestingly, some pathways related to cell fate commitment and specification (*P*-adjusted = 0.003) were up-regulated in the plate samples, represented by the over expression of *FZD7*, *SFRP2* and *SOX9* genes. The mesenchymal stem cells (MSCs) are the common precursor of adipocytes and osteoblasts and, therefore, the commitment of MSCs to adipocytes or osteoblasts has been especially implicated in abnormal bone remodeling diseases.

Finally, other processes related to the regulation of mitotic cell cycle and, more specifically the G1/S transition, showed up-regulation in the affected samples (*P*-adjusted = 0.002).

GO terms enrichment analysis of genes with the highest expression imbalance (Log2FC > |1.5|) between knee and control skin showed significant up-regulation in processes related to growth factor response (MAPK, BMP, Wnt and PI3K signalling pathways), epithelial cell proliferation, differentiation, and migration; chondrocyte differentiation and endochondral ossification; as well as skeletal system development. Moreover, a dysregulation of pathways related to cell commitment, embryonic epithelial morphogenesis and fat cell differentiation and commitment was also detected (**Fig. S5B**; **Table S4**).

However, processes related to skeletal system development, ossification, chondrocyte differentiation and endochondral ossification, as well as processes related to bone remodeling and mineralization, were found to be significantly down-regulated in the buttock skin sample (**Fig. S5B**; **Table S4**).

3.3. Blood mRNA and miRNAs profile in affected and unaffected twins

After differential expression analysis of RNA-seq data from peripheral blood of the patient with severe POH and the asymptomatic twin (using the simulated replicates), we obtained 969 DEGs (probability > 0.95), from which 675 were protein-coding genes and 157 were lnc-RNAs. Of these 969 genes, 537 (55.4%) showed overexpression in the asymptomatic twin, while 432 (44.6%) were found overexpressed in the severely affected twin (**Table S5**).

Among the top DEGs with the highest-ranking probability values (probability >0.99), the severe phenotype showed under-expression in several genes encoding ribosomal proteins (*RPS27*, *RPL39*, *RPL7* and *RPL17*), genes encoding components of cytochrome C oxidase involved in the mitochondrial electron transport chain (*NDUFA4* and *COX7C*), transcription-related genes (*ZNF22* and *POLR2K*) and immune response genes *(LY96* and different interferon-induced genes such as *IFI44L*, *IFI44*, *IFI27* or *IFIT1*). Conversely, genes encoding for antimicrobial proteins (*PI3, DEFA3, DEFA4*) were among the top DEGs showing over-expression in the severe patient. Another interesting over-expressed gene was the *MMP9* (Matrix Metallopeptidase 9) which encodes for a protein that plays an important role in the proteolytic degradation of the extracellular matrix, and with a possible role in osteoclastic bone resorption. The gene with the highest ranking value was *PI3* (Peptidase Inhibitor 3), which encodes for a protein (Elafin) that prevents elastase-mediated proteolysis in tissues and was identified in the horny layers of the human epidermis from psoriatic patients (*45*). Another over-expressed gene was *CHI3L1* (Chitinase 3 Like 1), which is related to homeostatic processes in tissues and secreted by activated macrophages, chondrocytes, neutrophils, and synovial cells. This gene is involved in inflammation, tissue remodeling and associated with an increased fibrotic activity and many pathological conditions. We also found over-expression of genes related to platelet activation/attachment, such as *PF4*, *GP9*, *TREML1*

and *MYL9*, and others involved in cell cycle regulation, such as *SPDYC* (Speedy/RINGO Cell Cycle Regulator Family Member C).

Pathways analysis carried out on the 969 DEGs emerging from the comparison of the blood transcriptomes of the two sisters showed an over-representation of several GO terms, the most relevant ones being those related to immune response, inflammation, platelet degranulation and activation, as well as coagulation processes (**Fig. S6A; Table S6**). The under-represented terms include some processes related to cell-cell signalling pathways, cell differentiation and migration, or key pathways involved in the regulation of cartilage development and bone formation, such as the Wnt or MAPK pathways (**Fig. S6B)**.

Comparison of blood miRNAs profiles in the affected and unaffected twin resulted in five miRNA differentially expressed ($log_2FC > |1.5|$ and > 100 counts in any of the samples; highlighted in yellow in **Table S7**). All of them but one showed over-expression in the affected twin, with log_2FC values ranging from 1.66 to 2.09, whereas the under-expressed miRNAs displayed a $log_2FC = -1.55$ (**Table S7**). A total of 793 predicted targets were detected for the five candidate miRNAs, hsa-miR-106b-5p being the one with the highest number of target genes (n = 656), followed by hsa-miR-493-3p (n = 72) and hsa-miR-556-5p (n = 47). The miRNA that reported the fewest predicted mRNA interactions was hsa-miR-518f-3p, with only 6 target genes (**Table S8**).

Only the most robust interactions between miRNAs and target mRNAs were selected for the downstream GO over-representation analysis (aggregated rank value <5; **Table S8; Fig. S7A**), thus including a total of 61 predicted targets (hsa-miR-106b-5p = 2; hsa-miR-493-3p = 18; hsa-miR-556-5p = 23; hsa-miR-518f-3p = 6; hsa-miR-208b-5p = 12) (**Fig. S7B**). We only found GO terms association (P-adjusted <0.05) for miRNA hsa-miR-493-3p, which was found over-expressed in the affected twin (**Table S9**, **Fig. S7C**). Significant terms obtained were related to cell fate commitment regulation (GO:0010454; GO:0010453) and regulation of transmembrane receptor protein serine/threonine kinase signaling pathway (GO:0090101; GO:0090092).

4. Additional Discussion

4.1. Dysregulation of cell dynamics-related processes

GNAS encodes the Gαs stimulatory subunit of heterotrimeric G-proteins, which is involved in the activation of intracellular signalling cascades in response to external stimuli. Its most characterized function entails targeting the adenylyl cyclase, which generates the second messenger cAMP and, in turn, increases the activity of the protein kinase A (PKA). cAMP has been described as a critical regulator of osteoblast/adipocyte commitment, and increased levels of cAMP have been demonstrated to favor adipogenesis over osteogenesis (*46*). In contrast, *GNAS* inactivation enhances osteogenic activity of MSCs from the adipose tissue (*47*). However, the contribution of other tissues as source of MSCs to the HO, such as muscle or skin, cannot be disregarded. Some authors have recently highlighted the importance of the local microenvironment in the HO process taking place in POH (*25*). At a subcutaneous level, *GNAS* inactivation produces strong micro-environment and histological changes in the adipose tissue, promoting the recruitment of MSCs and the formation of the HO in this region (*25*). HO generation starts with the recruitment of precursor cells, proliferation, condensation and the subsequent cell commitment and differentiation (*48, 49*). Pathways enrichment analysis carried out in our samples found some significant up- and down-regulated processes related to local cell dynamics in heterotopic plates and the knee skin compared to respective control samples. The most significant pathways (*P*-adjusted < 0.01) pointed to the involvement of processes such as stem cell proliferation, cell migration pathways, and epithelial cell proliferation as well as pathways related to cell fate commitment and differentiation in knee skin and heterotopic bone samples (**Fig. 1D**).

4.2. Endochondral origin of the heterotopic ossifications

Heterotopic bone formation is highly complex in POH and involves many cell types and tissues. Based on histopathological studies, HO in POH is mainly formed through an intramembranous ossification, and the appearance of endochondral ossifications has been described as sporadic (*9*); this process however has not been studied at a molecular level so far. A recent study in *GNAS*-null mouse model mimicking the POH molecular condition suggested an

endochondral origin for the HO (*25*). Despite of the skin samples of our case study being collected from different anatomical regions (scapular and abdominal), we found them to show a very similar expression pattern. Transcriptomic profiles suggest that both tissue plates would have an endochondral origin, or at least show evidence of having active chondrogenesis, because genes showing the higher log_2FC values were closely related to cartilage development and chondrocyte differentiation (e.g. *SOX9*, *COMP* or *TGFB3*; **Fig. 1C; Fig. S3**). Thus, *SOX9* was found to be up-regulated in heterotopic bones. *SOX9* precisely regulates cell differentiation in growth plate chondrocytes (*50*) as well as adipogenic differentiation in MSCs (*51*). In addition, we found an up-regulation of the gene Peroxisome Proliferative-Activated Receptor Gamma Coactivator 1 Alpha (*PPARGC1A*; also known as *PGC1A*), which has been found to be over-expressed during chondrogenesis of MSCs (*52*). *PPARGC1A* is an important regulator of many biological processes, such as mitochondrial bio-genesis, thermogenesis and lipid metabolism (*53*), but it can also act as co-activator for *SOX9* and co-regulate chondrogenesis after stimulation through TGF-β. In agreement with this observation, *TGFB3*, which is an important chondrogenic inductor, was also found over-expressed in both heterotopic samples (log_2FC of 1.57 [abdominal] and 1.62 [scapular]) and the affected skin sample (log_2FC = 1.72) when compared to the controls. Co-over-expression of *PPARGC1A* and *SOX9* can activate the expression of other ECM chondrogenic proteins (*COMP*, aggrecan) (*52*). However, although *SOX9* over-expression is an enhancer of chondrogenic differentiation in MSCs, it also slows down the hypertrophic pathway through the down-regulation of Wnt pathway to maintain the chondrogenic state (*54, 55*).

The cartilage matrix generated during endochondral bone formation is composed of different ECM proteins, the most important being proteoglycans aggrecan, decorin, biglycan, fibromodulin, perlecan and collagen isotypes Type II, Type IX and Type XI (*56*). These proteins appear at different timepoints of bone formation. Thus, for instance, the primitive cartilaginous matrix is rich in collagen Type II and Type XI; while at a more advance stage, chondrocytes become hypertrophied, expressing collagen Type X instead, and activating mineralization and the degradation of the surrounding ECM. Although Type X collagen was not included in the expression panel, we found similar expression

levels of *COL2A1* gene (which produces Type II collagen) in heterotopic bone samples and control, but under-expression of *COL11A2* gene (which produces collagen Type XI), suggesting a more advanced stage of the chondrogenic phenotype.

Chondrogenic ECM also includes the multifunctional family of proteins thrombospondins; recent studies suggest that thrombospondins have different and sequential roles during bone formation. The most studied one from cartilage is thrombospondin-5 (Cartilage Oligomeric Matrix Protein), which is encoded by the *COMP* gene (also called *THBS5*). Thrombospondin-5 is present across all the transient cartilage matrix (*57*) and it plays a critical role in cartilage bone matrix organization (*58*). Mutations in *COMP* can alter cartilage and bone formation, leading to reduced bone mass mineralization and the development of skeletal disorders (*59*). Instead, Thrombospondin-4, encoded by *THBS4* gene, is the only protein with angiogenic potential within this protein family. Thus, while *COMP* is expressed during all the chondrogenic phases, *THBS4* expresses predominantly in hypertrophic chondrocytes (*60*). Our findings indicate that *COMP* was the most over-expressed gene in the bone samples ($log_2FC > 6$) when compared to control sample, pointing again to a probable endochondral origin of these plates (**Fig. 1C; Fig. S3**). Also, *THBS4* showed different up-regulation levels between plate samples (Log_2FC of 0.98 and 3.15 in abdominal and scapular plates, respectively), suggesting a dominant endochondral function in the plates.

Other proteins with an important role in ECM remodeling are the zinc-dependent metalloproteinases (MMPs). These proteins can degrade all types of ECM protein components involved in many biological processes, including skeletal development, bone growth and remodeling. In line with a dominant endochondral ossification process, *MMP7* (matrilysin) gene, an MMP proteinase with a role in chondrogenesis (*61, 62*), was found highly expressed in heterotopic bones, showing log_2FC values > 5 in both scapular and abdominal plates.

After the differentiation through both endochondral and intramembranous processes, osteoblasts mainly produce Type I collagen (encoded by e.g. *COL1A1*, *COL1A2* genes), which is not expressed in mature chondrocytes and represents more than 90% of bone (*63*). During osteoblast differentiation, *RUNX2* enhances the expression of major bone matrix genes including *COL1A1*,

COL1A2, *SPP1* (secreted phosphoprotein 1), *IBSP* (integrin-binding sialoprotein), *BGLAP* (osteocalcin) and *FN1* (fibronectin 1) (*64*). Expression values of bone matrix makers included in the panel were lower in the buttock skin sample than those from the skin control sample.

4.3. Dysregulation of Wnt pathway

Wnt pathway is involved in the differentiation of MSCs to chondrocytes and osteoblasts, but the specifics of the mechanisms involved are still debated. A prolonged low level activation of the canonical Wnt pathway is necessary to trigger chondrogenesis (*65*), inducing *SOX9* expression and activating MSCs differentiation, while increased levels of Wnt lead to chondrocyte hypertrophy (*66*) but can have a detrimental effect on chondrogenesis (*65*). Molecular regulation of Wnt requires fine tuning, is complex and many of its features are still unknown, particularly regarding the balance between canonical and non-canonical Wnt pathways and the role of specific ligands and receptors. In the absence of the Wnt ligands, cytoplasmic β-catenin is phosphorylated by an intracellular protein complex composed of glycogen synthase kinase 3β (*GSK3B*), the scaffolding protein axin (*AXIN1*) and the tumor suppressor adenomatous polyposis coli (*APC*), and subsequently ubiquitinylated and degraded by the proteasome (*67*). These intracellular inhibitors showed similar expression levels in controls and patient samples in both HO and skin tissues. Extracellular antagonist of Wnt signaling can also negatively regulate the pathway by binding Wnt proteins to block their union to Fzd and LRP5/6 receptors, such as secreted Fz-related-proteins (SFRPs) and Wnt inhibitory factor 1 (*WIF1*), or by direct binding to Fdz and LRP5/6 receptors, such as dickkopf (*DKK*) and sclerostin (*SOST*) (*68, 69*). We did not detect expression of *SOST* in any of the tissues analyzed, and only a slight over-expression of *DKK1* and *DKK2* in the HO samples. However, extracellular Wnt inhibitors were found strongly up-regulated in both heterotopic plates (*SFRP1*, *SFRP2*), and knee skin (*SFRP1*, *SFRP2, SFRP4*), with respect to control samples, suggesting a Wnt signalling drop-off. This would be supported by the fact that downstream target genes (*LEF1*, *TCF7L1*, *TCF3*, *CCND1*, *AXIN2*, *MYC*) did not show signals of over-expression in these samples. In contrast, *WIF1* antagonist was under-regulated in the HO with respect to control bone (*70*). However, the role of SFRPs and other Wnt modulators is still poorly understood

because it is complex, highly dynamic, and probably influenced by the cellular context and differentiation stage. Indeed, it has been shown that SFRPs can also act as Wnt agonist; for instance, *SFRP2* has been shown to induce osteogenic differentiation by activating Wnt canonical pathway (*71*). In addition, SFRPs could play an additional role effectively expanding the signalling area of Wnt pathway after SPFR-Wnts interactions (*72*). To add a further degree of complexity, activation of Wnt pathway also triggers some Wnt inhibitory molecules (i.e. *AXIN2*); therefore, Wnt-related inferences based on experimental findings are not obvious and should be interpreted with caution.

GNAS inactivation weakened Wnt canonical signaling and the commitment of MSCs to osteoblasts. Thus, some authors indicate that an up-regulation of the Hedgehog signaling may be sufficient to induce HO (*15*). Nevertheless, genes involved in Hh pathway (membrane receptors *PTCH1* and *SMO*; transcriptional activators *GLI1* and *GLI3*; co-receptors *GAS1* and *LRP2*) did not show significant differences in expression with respect to control samples in any of the tissues analyzed.

4.4. Local TGF-β involvement

Bone formation, remodeling and repair processes are under the regulation of TGF-β (*73*). TGF-β is required in all phases of chondrogenesis, mesenchymal condensation, chondrocyte proliferation, ECM deposition and, finally, differentiation (*21*). Related to this, the abovementioned up-regulation of *TGFB3* gene observed in the HO and the affected skin samples is particularly relevant, since TGF-β3 has been demonstrated to be a potent inductor of chondrogenic differentiation in adipose derived stem cells (*74, 75*). Moreover, an induction of Wnt signalling can lead to a chondrogenic differentiation process in the presence of TGF-β3, but hindering chondrocyte hypertrophy and calcification (*76*). TGF-β also plays an important role in HO, as high levels of active TGF-β induces MSCs migration and recruitment, initiating HO process and angiogenesis. In line with this, administration of inhibitors of TGF-β signaling has been proposed as a treatment against HO (*77, 78*). In this line, we identified TGF-β as the most significant altered pathway in our heterotopic bone and knee skin samples when compared to control. In contrast, TGF-β pathways were significantly down-regulated in the buttock skin sample, showing the lowest *P*-adjusted value.

Interestingly, *LIFR* was up-regulated in the affected skin sample ($Log_2FC = 3.13$; Fig. 5B; **Fig. S3**) but not in the other tissues. *LIF* and *LIFR* can be produced by fibroblasts and chondrocytes. TGF-β stimulates osteoblast differentiation and also the expression of *LIF* (*79*), thus, over-activation of TGF-β together with the over-expression of *LIFR* by the cells from knee skin sample could help to establish a hypersensitive local microenvironment that would contribute to a greater and more efficient response to *LIF*. In parallel, some studies have claimed that *LIF* in cell culture assays enhances the expression of *SPP1* (*80*), which was found up-regulated in the same sample.

Other pathways related to TGF-β regulation and activation were also significantly dysregulated, for instance SMAD, which is the canonical signaling pathway directly activated by the TGF-β, and morphogenetic protein (BMP) pathways, a key regulatory role in bone homeostasis (*81*). *GNAS* inactivation stimulates BMP-SMAD signaling *via* cAMP in MSCs located in the soft connective tissue inducing HO formation (*46*). Both BMP and SMAD related pathways were found significantly dysregulated in HO and the knee skin. of the BMP proteins, which are part of the TGF-β family, *BMP5* was found strongly under-regulated in heterotopic bone samples ($Log_2FC < -4.5$). *BMP5* is an important regulator of chondrocyte proliferation and differentiation by promoting chondrocyte hypertrophy and matrix maturation (*82, 83*). However, *BMP5* silencing decreases ECM degradation and increases ECM synthesis, pointing to a regulatory role of *BMP5* in chondrocyte senescence and apoptosis (*84*).

TGF-β can play a dual role. In normal epithelium it acts as a tumor suppressor through the regulation of cell proliferation, the activation of apoptosis and the inhibition of cell immortalization. However, in aggressive and invasive tumors, TGF-β has a pro-oncogenic activity, affecting tumor microenvironment, promoting cell stemness, cell motility, epithelia to mesenchymal transition (EMT), angiogenesis, and inhibiting immune cell functions (*85*). EMT cannot be disregarded as one of the possible mechanisms that promotes the appearance, growth and dispersion of the heterotopic lesions. New pluripotent EMT-mesenchymal cells are highly motile and have the capacity to invade different tissues. Studies in FOP have highlighted the importance of endothelial to mesenchymal transition in the initial stage of HO lesion formation (*86-88*).

Moreover, dysregulation of some genes pointed in the same direction, for instance *MMP7* can enable cells to migrate and invade surrounding tissues facilitating tumor progression in cancer (*89*). *MMP7* is also over-expressed in many types of tumor cells (*90, 91*), probably enhancing tumor progression through de apoptosis inhibition in cancer cells (*92*), reducing cell adhesion (*93*) and inducing angiogenesis (*94*). It has been suggested that down-regulation of *BMP5* may be related to metastatic events in different type of cancers (*95, 96*), and also lower expression of *WIF1* was associated with lung cancer (*97*).

4.5. Systemic signals of severe POH

At systemic level, the analysis of blood gene expression patterns in non-affected and affected twins revealed some DEGs and pathways differentiating both phenotypes. Among the significantly over-represented categories, we found routes related to immune response, inflammation, extracellular matrix organization and cell adhesion, motility, and proliferation. Interestingly, we also found a significant over-representation of genes involved in platelet activation. *In vitro* studies have demonstrated that, after activation, platelets can release inflammatory factors and other molecules that enhance the MSCs differentiation and proliferation, fibroblast proliferation, angiogenesis, and ECM deposition (*98, 99*); in fact, platelet concentrates have been tested in different contexts due to their capacity to improve bone formation and healing. Locally, Platelet Derived Growth Factor Receptor Alpha receptor gene (*PDGFRB*) showed up-regulation in the affected skin (**Fig. S3**). *PDGFRB* is expressed in mesenchymal cells and osteoblasts, and the expression of PDGFRs is increased in response to an inflammatory process (i.e. fractures) (*100*). On the other hand, some significantly depleted pathways related to osseous metabolism were also detected, including Wnt and MAPK signalling.

Analysis of systemic miRNA profiles yielded five top miRNAs differentially expressed between the twins. MiRNAs regulate mRNA expression by binding to a specific mRNA at the 3′-UTR to induce translational repression. The miR-106b-5p miRNA was the only one under-expressed in the affected twin. Interestingly, it has been shown that miR-106b-5p can inhibit the process of osteogenic differentiation and bone formation in vivo by targeting SMAD5 (*101*). Therefore, a systemic under-expression of miR-106b-5p could lead to an activation of the

osteogenic activity via SMAD5. In addition, over-expressed miR-493-3p could be involved in the regulation of cell fate commitment through the interaction with Dickkopf-related protein 1 (*DKK1*) and Sclerostin domain-containing protein 1 (*SOSTDC1*). *DKK1* gene is a secreted Wnt pathway antagonist and plays an important role in bone metabolism regulation. In the same way, *SOSTDC1* is a secreted inhibitor of BMP pathway and can also regulate the Wnt signalling by inhibiting or enhancing its activity (*102*).

5. Study limitations

The main limitation of the present study is related to the limited sample size. Such circumstance is common in molecular studies dealing with rare diseases due not only to the very low incidence of the condition (even more extreme in ultra-rare disease), but also because: *i*) the onset of many rare diseases is at pediatric age and often have bad prognosis, even leading to patient death at younger ages; *ii*) misdiagnosis derived from phenotypic heterogeny of the disease; and *iii*) the geographic spread of the affected subjects. In our case study, having control tissue samples represents another challenge due to the unusual biological source of the target tissues. In the case of the affected twin, skin and plate samples availability was subjected to the palliative surgeries carried out to the affected patient, whereas it was not possible to collect tissue samples from the asymptomatic healthy twin. Despite the limitations, the extraordinary characteristics of the twin case analyzed in the present study offered an unique opportunity to shed light on an extremely complex disease condition, and it could also help illuminate other *GNAS* and non *GNAS*-related disorders presenting HOs.

6. References

1. F. S. Kaplan *et al.*, Progressive osseous heteroplasia: a distinct developmental disorder of heterotopic ossification. Two new case reports and follow-up of three previously reported cases. *J Bone Joint Surg Am* **76**, 425-436 (1994).
2. F. S. Kaplan, E. M. Shore, Progressive osseous heteroplasia. *J Bone Miner Res* **15**, 2084-2094 (2000).

3. L. S. Weinstein, S. Yu, D. R. Warner, J. Liu, Endocrine manifestations of stimulatory G protein alpha-subunit mutations and the role of genomic imprinting. *Endocr Rev* **22**, 675-705 (2001).
4. V. Syrovatkina, K. O. Alegre, R. Dey, X. Y. Huang, Regulation, Signaling, and Physiological Functions of G-Proteins. *J Mol Biol* **428**, 3850-3868 (2016).
5. G. Mantovani *et al.*, Biallelic expression of the Gsalpha gene in human bone and adipose tissue. *J Clin Endocrinol Metab* **89**, 6316-6319 (2004).
6. M. Bastepe, The GNAS Locus: Quintessential Complex Gene Encoding Gsalpha, XLalphas, and other Imprinted Transcripts. *Curr Genomics* **8**, 398-414 (2007).
7. G. Kelsey, Imprinting on chromosome 20: tissue-specific imprinting and imprinting mutations in the GNAS locus. *Am J Med Genet C Semin Med Genet* **154C**, 377-386 (2010).
8. E. M. Shore *et al.*, Paternally inherited inactivating mutations of the GNAS1 gene in progressive osseous heteroplasia. *N Engl J Med* **346**, 99-106 (2002).
9. N. S. Adegbite, M. Xu, F. S. Kaplan, E. M. Shore, R. J. Pignolo, Diagnostic and mutational spectrum of progressive osseous heteroplasia (POH) and other forms of GNAS-based heterotopic ossification. *Am J Med Genet A* **146A**, 1788-1796 (2008).
10. E. M. Shore, F. S. Kaplan, Insights from a rare genetic disorder of extra-skeletal bone formation, fibrodysplasia ossificans progressiva (FOP). *Bone* **43**, 427-433 (2008).
11. R. Happle, Progressive osseous heteroplasia is not a Mendelian trait but a type 2 segmental manifestation of GNAS inactivation disorders: A hypothesis. *Eur J Med Genet* **59**, 290-294 (2016).
12. R. Happle, The categories of cutaneous mosaicism: A proposed classification. *Am J Med Genet A* **170A**, 452-459 (2016).
13. F. S. Kaplan, R. J. Pignolo, E. M. Shore, The FOP metamorphogene encodes a novel type I receptor that dysregulates BMP signaling. *Cytokine Growth Factor Rev* **20**, 399-407 (2009).

14. E. M. Shore *et al.*, A recurrent mutation in the BMP type I receptor ACVR1 causes inherited and sporadic fibrodysplasia ossificans progressiva. *Nat Genet* **38**, 525-527 (2006).
15. J. B. Regard *et al.*, Activation of Hedgehog signaling by loss of GNAS causes heterotopic ossification. *Nat Med* **19**, 1505-1512 (2013).
16. J. Yang, P. Andre, L. Ye, Y. Z. Yang, The Hedgehog signalling pathway in bone formation. *Int J Oral Sci* **7**, 73-79 (2015).
17. H. Hojo *et al.*, Hedgehog-Gli activators direct osteo-chondrogenic function of bone morphogenetic protein toward osteogenesis in the perichondrium. *J Biol Chem* **288**, 9924-9932 (2013).
18. R. Baron, M. Kneissel, WNT signaling in bone homeostasis and disease: from human mutations to treatments. *Nat Med* **19**, 179-192 (2013).
19. Y. Wang *et al.*, Wnt and the Wnt signaling pathway in bone development and disease. *Front Biosci (Landmark Ed)* **19**, 379-407 (2014).
20. K. Maeda *et al.*, The Regulation of Bone Metabolism and Disorders by Wnt Signaling. *Int J Mol Sci* **20**, (2019).
21. M. Wu, G. Chen, Y. P. Li, TGF-β and BMP signaling in osteoblast, skeletal development, and bone formation, homeostasis and disease. *Bone Res* **4**, 16009 (2016).
22. A. S. Chagin, Effectors of mTOR-autophagy pathway: targeting cancer, affecting the skeleton. *Curr Opin Pharmacol* **28**, 1-7 (2016).
23. H. Wu *et al.*, Bone Size and Quality Regulation: Concerted Actions of mTOR in Mesenchymal Stromal Cells and Osteoclasts. *Stem Cell Reports* **8**, 1600-1616 (2017).
24. K. K. Mak, M. H. Chen, T. F. Day, P. T. Chuang, Y. Yang, Wnt/beta-catenin signaling interacts differentially with Ihh signaling in controlling endochondral bone and synovial joint formation. *Development* **133**, 3695-3707 (2006).
25. N. Brewer, J. T. Fong, D. Zhang, G. Ramaswamy, E. M. Shore, Inactivation Alters Subcutaneous Tissues in Progression to Heterotopic Ossification. *Front Genet* **12**, 633206 (2021).
26. Q. Cong *et al.*, A self-amplifying loop of YAP and SHH drives formation and expansion of heterotopic ossification. *Sci Transl Med* **13**, (2021).

27. C. Kan *et al.*, Conserved signaling pathways underlying heterotopic ossification. *Bone* **109**, 43-48 (2018).
28. A. W. James, Review of Signaling Pathways Governing MSC Osteogenic and Adipogenic Differentiation. *Scientifica (Cairo)* **2013**, 684736 (2013).
29. A. J. Justicia-Grande *et al.*, Case Report: Two Monochorionic Twins With a Critically Different Course of Progressive Osseus Heteroplasia. *Front Pediatr* **9**, 662669 (2021).
30. M. Cebey-López *et al.*, Case Report: Everolimus reduced bone turnover markers but showed no clinical benefit in a patient with severe progressive osseous heteroplasia. *Front Pediatr* **10**, 936780 (2022).
31. J. Vandesompele *et al.*, Accurate normalization of real-time quantitative RT-PCR data by geometric averaging of multiple internal control genes. *Genome Biol* **3**, RESEARCH0034 (2002).
32. A. Gómez-Carballa *et al.*, A multi-tissue study of immune gene expression profiling highlights the key role of the nasal epithelium in COVID-19 severity. *Environ Res*, 112890 (2022).
33. Z. Gu, R. Eils, M. Schlesner, Complex heatmaps reveal patterns and correlations in multidimensional genomic data. *Bioinformatics* **32**, 2847-2849 (2016).
34. R. C. Team, R: A Language and Environment for Statistical Computing. *Vienna, Austria* https://www.R-project.org, (2019).
35. A. Salas *et al.*, Strong down-regulation of glycophorin genes: A host defense mechanism against rotavirus infection. *Infect Genet Evol* **44**, 403-411 (2016).
36. A. M. Bolger, M. Lohse, B. Usadel, Trimmomatic: a flexible trimmer for Illumina sequence data. *Bioinformatics* **30**, 2114-2120 (2014).
37. A. Dobin *et al.*, STAR: ultrafast universal RNA-seq aligner. *Bioinformatics* **29**, 15-21 (2013).
38. H. Li *et al.*, The Sequence Alignment/Map format and SAMtools. *Bioinformatics* **25**, 2078-2079 (2009).
39. B. Li, C. N. Dewey, RSEM: accurate transcript quantification from RNA-Seq data with or without a reference genome. *BMC Bioinformatics* **12**, 323 (2011).

40. S. Tarazona *et al.*, Data quality aware analysis of differential expression in RNA-seq with NOISeq R/Bioc package. *Nucleic Acids Res* **43**, e140 (2015).
41. M. D. Young, M. J. Wakefield, G. K. Smyth, A. Oshlack, Gene ontology analysis for RNA-seq: accounting for selection bias. *Genome Biol* **11**, R14 (2010).
42. P. M, S. TI. (2021).
43. G. Yu, L. G. Wang, Y. Han, Q. Y. He, clusterProfiler: an R package for comparing biological themes among gene clusters. *OMICS* **16**, 284-287 (2012).
44. G. Yu, enrichplot: Visualization of Functional Enrichment Result. *R package version 1.6.1*, (2019).
45. O. Wiedow, J. M. Schröder, H. Gregory, J. A. Young, E. Christophers, Elafin: an elastase-specific inhibitor of human skin. Purification, characterization, and complete amino acid sequence. *J Biol Chem* **265**, 14791-14795 (1990).
46. S. Zhang, F. S. Kaplan, E. M. Shore, Different roles of GNAS and cAMP signaling during early and late stages of osteogenic differentiation. *Horm Metab Res* **44**, 724-731 (2012).
47. R. J. Pignolo *et al.*, Heterozygous inactivation of Gnas in adipose-derived mesenchymal progenitor cells enhances osteoblast differentiation and promotes heterotopic ossification. *J Bone Miner Res* **26**, 2647-2655 (2011).
48. C. Hartmann, Transcriptional networks controlling skeletal development. *Curr Opin Genet Dev* **19**, 437-443 (2009).
49. G. L. Galea, M. R. Zein, S. Allen, P. Francis-West, Making and shaping endochondral and intramembranous bones. *Dev Dyn* **250**, 414-449 (2021).
50. P. Dy *et al.*, Sox9 directs hypertrophic maturation and blocks osteoblast differentiation of growth plate chondrocytes. *Dev Cell* **22**, 597-609 (2012).
51. S. Stöckl *et al.*, Sox9 modulates cell survival and adipogenic differentiation of multipotent adult rat mesenchymal stem cells. *J Cell Sci* **126**, 2890-2902 (2013).

52. Y. Kawakami *et al.*, Transcriptional coactivator PGC-1alpha regulates chondrogenesis via association with Sox9. *Proc Natl Acad Sci U S A* **102**, 2414-2419 (2005).
53. P. Puigserver, B. M. Spiegelman, Peroxisome proliferator-activated receptor-gamma coactivator 1 alpha (PGC-1 alpha): transcriptional coactivator and metabolic regulator. *Endocr Rev* **24**, 78-90 (2003).
54. H. Akiyama *et al.*, Interactions between Sox9 and beta-catenin control chondrocyte differentiation. *Genes Dev* **18**, 1072-1087 (2004).
55. L. Topol, W. Chen, H. Song, T. F. Day, Y. Yang, Sox9 inhibits Wnt signaling by promoting beta-catenin phosphorylation in the nucleus. *J Biol Chem* **284**, 3323-3333 (2009).
56. C. J. Malemud, N. Islam, T. M. Haqqi, Pathophysiological mechanisms in osteoarthritis lead to novel therapeutic strategies. *Cells Tissues Organs* **174**, 34-48 (2003).
57. M. D. Briggs *et al.*, Pseudoachondroplasia and multiple epiphyseal dysplasia due to mutations in the cartilage oligomeric matrix protein gene. *Nat Genet* **10**, 330-336 (1995).
58. J. N. Schulz *et al.*, COMP-assisted collagen secretion--a novel intracellular function required for fibrosis. *J Cell Sci* **129**, 706-716 (2016).
59. F. Coustry *et al.*, Mutant cartilage oligomeric matrix protein (COMP) compromises bone integrity, joint function and the balance between adipogenesis and osteogenesis. *Matrix Biol* **67**, 75-89 (2018).
60. E. Andrés Sastre *et al.*, Spatiotemporal distribution of thrombospondin-4 and -5 in cartilage during endochondral bone formation and repair. *Bone* **150**, 115999 (2021).
61. F. Djouad *et al.*, Microenvironmental changes during differentiation of mesenchymal stem cells towards chondrocytes. *Arthritis Res Ther* **9**, R33 (2007).
62. C. S. Bahney, C. W. Hsu, J. U. Yoo, J. L. West, B. Johnstone, A bioresponsive hydrogel tuned to chondrogenesis of human mesenchymal stem cells. *FASEB J* **25**, 1486-1496 (2011).
63. P. Gómez-Picos, B. F. Eames, On the evolutionary relationship between chondrocytes and osteoblasts. *Front Genet* **6**, 297 (2015).

64. T. Komori, Regulation of bone development and extracellular matrix protein genes by RUNX2. *Cell Tissue Res* **339**, 189-195 (2010).
65. N. P. Schizas *et al.*, Inhibition versus activation of canonical Wnt-signaling, to promote chondrogenic differentiation of Mesenchymal Stem Cells. A review. *Orthop Rev (Pavia)* **13**, 27098 (2021).
66. F. Fagotto, Looking beyond the Wnt pathway for the deep nature of β-catenin. *EMBO Rep* **14**, 422-433 (2013).
67. J. L. Stamos, W. I. Weis, The β-catenin destruction complex. *Cold Spring Harb Perspect Biol* **5**, a007898 (2013).
68. Y. Kawano, R. Kypta, Secreted antagonists of the Wnt signalling pathway. *J Cell Sci* **116**, 2627-2634 (2003).
69. A. Filipovich, I. Gehrke, S. J. Poll-Wolbeck, K. A. Kreuzer, Physiological inhibitors of Wnt signaling. *Eur J Haematol* **86**, 453-465 (2011).
70. T. M. Yang *et al.*, WIF-1 promoter region hypermethylation as an adjuvant diagnostic marker for non-small cell lung cancer-related malignant pleural effusions. *J Cancer Res Clin Oncol* **135**, 919-924 (2009).
71. L. F. deCastro *et al.*, Secreted frizzled related-protein 2 (Sfrp2) deficiency decreases adult skeletal stem cell function in mice. *Bone Res* **9**, 49 (2021).
72. Y. Mii, M. Taira, Secreted Frizzled-related proteins enhance the diffusion of Wnt ligands and expand their signalling range. *Development* **136**, 4083-4088 (2009).
73. Y. Tang *et al.*, TGF-beta1-induced migration of bone mesenchymal stem cells couples bone resorption with formation. *Nat Med* **15**, 757-765 (2009).
74. S. Zhu *et al.*, Programmed Application of Transforming Growth Factor β3 and Rac1 Inhibitor NSC23766 Committed Hyaline Cartilage Differentiation of Adipose-Derived Stem Cells for Osteochondral Defect Repair. *Stem Cells Transl Med* **3**, 1242-1251 (2014).
75. A. Technau, K. Froelich, R. Hagen, N. Kleinsasser, Adipose tissue-derived stem cells show both immunogenic and immunosuppressive properties after chondrogenic differentiation. *Cytotherapy* **13**, 310-317 (2011).
76. P. Tanthaisong *et al.*, Enhanced Chondrogenic Differentiation of Human Umbilical Cord Wharton's Jelly Derived Mesenchymal Stem Cells by GSK-3 Inhibitors. *PLoS One* **12**, e0168059 (2017).

77. D. Mao, X. Pan, Y. Rui, F. Li, Matrine attenuates heterotopic ossification by suppressing TGF-β induced mesenchymal stromal cell migration and osteogenic differentiation. *Biomed Pharmacother* **127**, 110152 (2020).
78. X. Wang *et al.*, Inhibition of overactive TGF-β attenuates progression of heterotopic ossification in mice. *Nat Commun* **9**, 551 (2018).
79. N. A. Sims, R. W. Johnson, Leukemia inhibitory factor: a paracrine mediator of bone metabolism. *Growth Factors* **30**, 76-87 (2012).
80. M. Noda, R. L. Vogel, D. M. Hasson, G. A. Rodan, Leukemia inhibitory factor suppresses proliferation, alkaline phosphatase activity, and type I collagen messenger ribonucleic acid level and enhances osteopontin mRNA level in murine osteoblast-like (MC3T3E1) cells. *Endocrinology* **127**, 185-190 (1990).
81. G. Sánchez-Duffhues, C. Hiepen, P. Knaus, P. Ten Dijke, Bone morphogenetic protein signaling in bone homeostasis. *Bone* **80**, 43-59 (2015).
82. S. J. Snelling, P. A. Hulley, J. Loughlin, BMP5 activates multiple signaling pathways and promotes chondrogenic differentiation in the ATDC5 growth plate model. *Growth Factors* **28**, 268-279 (2010).
83. G. Mailhot *et al.*, BMP-5 expression increases during chondrocyte differentiation in vivo and in vitro and promotes proliferation and cartilage matrix synthesis in primary chondrocyte cultures. *J Cell Physiol* **214**, 56-64 (2008).
84. Y. Shao *et al.*, BMP5 silencing inhibits chondrocyte senescence and apoptosis as well as osteoarthritis progression in mice. *Aging (Albany NY)* **13**, 9646-9664 (2021).
85. J. J. Lebrun, The Dual Role of TGFβ in Human Cancer: From Tumor Suppression to Cancer Metastasis. *ISRN Mol Biol* **2012**, 381428 (2012).
86. S. Agarwal *et al.*, Local and Circulating Endothelial Cells Undergo Endothelial to Mesenchymal Transition (EndMT) in Response to Musculoskeletal Injury. *Sci Rep* **6**, 32514 (2016).
87. J. Zhang *et al.*, Neurotrophin-3 acts on the endothelial-mesenchymal transition of heterotopic ossification in rats. *J Cell Mol Med* **23**, 2595-2609 (2019).

88. D. Medici *et al.*, Conversion of vascular endothelial cells into multipotent stem-like cells. *Nat Med* **16**, 1400-1406 (2010).
89. C. Yan, D. D. Boyd, Regulation of matrix metalloproteinase gene expression. *J Cell Physiol* **211**, 19-26 (2007).
90. Y. Adachi *et al.*, Contribution of matrilysin (MMP-7) to the metastatic pathway of human colorectal cancers. *Gut* **45**, 252-258 (1999).
91. H. Yamamoto *et al.*, Expression of matrix metalloproteinases and tissue inhibitors of metalloproteinases in human pancreatic adenocarcinomas: clinicopathologic and prognostic significance of matrilysin expression. *J Clin Oncol* **19**, 1118-1127 (2001).
92. W. S. Wang, P. M. Chen, H. S. Wang, W. Y. Liang, Y. Su, Matrix metalloproteinase-7 increases resistance to Fas-mediated apoptosis and is a poor prognostic factor of patients with colorectal carcinoma. *Carcinogenesis* **27**, 1113-1120 (2006).
93. D. C. von Bredow, R. B. Nagle, G. T. Bowden, A. E. Cress, Cleavage of beta 4 integrin by matrilysin. *Exp Cell Res* **236**, 341-345 (1997).
94. M. Ii, H. Yamamoto, Y. Adachi, Y. Maruyama, Y. Shinomura, Role of matrix metalloproteinase-7 (matrilysin) in human cancer invasion, apoptosis, growth, and angiogenesis. *Exp Biol Med (Maywood)* **231**, 20-27 (2006).
95. M. A. Karim *et al.*, A Multi-Omics Analysis of Bone Morphogenetic Protein 5 (BMP5) mRNA Expression and Clinical Prognostic Outcomes in Different Cancers Using Bioinformatics Approaches *Biomedicines* **8**, (2020).
96. B. Bragdon *et al.*, Bone morphogenetic proteins: a critical review. *Cell Signal* **23**, 609-620 (2011).
97. Q. Tang *et al.*, WIF-1 gene inhibition and Wnt signal transduction pathway activation in NSCLC tumorigenesis. *Oncol Lett* **13**, 1183-1188 (2017).
98. L. C. Visser *et al.*, Growth factor-rich plasma increases tendon cell proliferation and matrix synthesis on a synthetic scaffold: an in vitro study. *Tissue Eng Part A* **16**, 1021-1029 (2010).
99. C. Doucet *et al.*, Platelet lysates promote mesenchymal stem cell expansion: a safety substitute for animal serum in cell-based therapy applications. *J Cell Physiol* **205**, 228-236 (2005).

100. C. H. Heldin, B. Westermark, Mechanism of action and in vivo role of platelet-derived growth factor. *Physiol Rev* **79**, 1283-1316 (1999).
101. T. Fang, Q. Wu, L. Zhou, S. Mu, Q. Fu, miR-106b-5p and miR-17-5p suppress osteogenic differentiation by targeting Smad5 and inhibit bone formation. *Exp Cell Res* **347**, 74-82 (2016).
102. K. A. Clausen *et al.*, SOSTDC1 differentially modulates Smad and beta-catenin activation and is down-regulated in breast cancer. *Breast Cancer Res Treat* **129**, 737-746 (2011).

7. Legend to the supplementary tables

Table S1. Description of the samples included in the analysis.

Table S2. Normalized expression of heterotopic bone samples and bone from healthy control. The log_2FC values between comparisons are also provided.

Table S3. Normalized expression of skin samples from the patient and skin from healthy control. The log_2FC values between comparisons are also provided.

Table S4. Pathway analysis results from heterotopic ossification and skin samples.

Table S5. Differentially expressed genes between blood samples from the affected and the non-affected POH patients considering a probability cut-off point of 0.99.

Table S6. Pathways enrichment analysis from DEGs in blood samples showing the over- (A) and under-represented (B) significant terms.

Table S7. Normalized expression of blood miRNAs from affected and non-affected twin and log_2FC values between comparison of both samples.

Table S8. miRNAs target genes predicted from miRNAs databases. Target genes with aggregated rank value <5 were selected for downstream pathways analysis (highlighted in yellow).

Table S9. Pathways enrichment analysis results using the top predicted target genes from the 5 miRNAs differentially expressed as input.

8. Supplementary Figures

Fig. S1. (A) Tridimensional TC of lower limbs performed in the same year as the collection of the skin samples. The picture shows the calcification in the right leg, and the ankylosis, asymmetry, and tibial combing of the left leg. (B) Left: Histological sample of cutaneous and subcutaneous tissue collected from the left leg stained with hematoxylin and eosin (HE). Extensive ossification at deep dermal level and hypodermis can be observed. Sweat gland is included (40x). Top-right: Bone trabeculae with variable wells separated by adipose tissue (100x). Bottom-right: ossification integrated in muscle, surrounded by adipose tissue (40x).

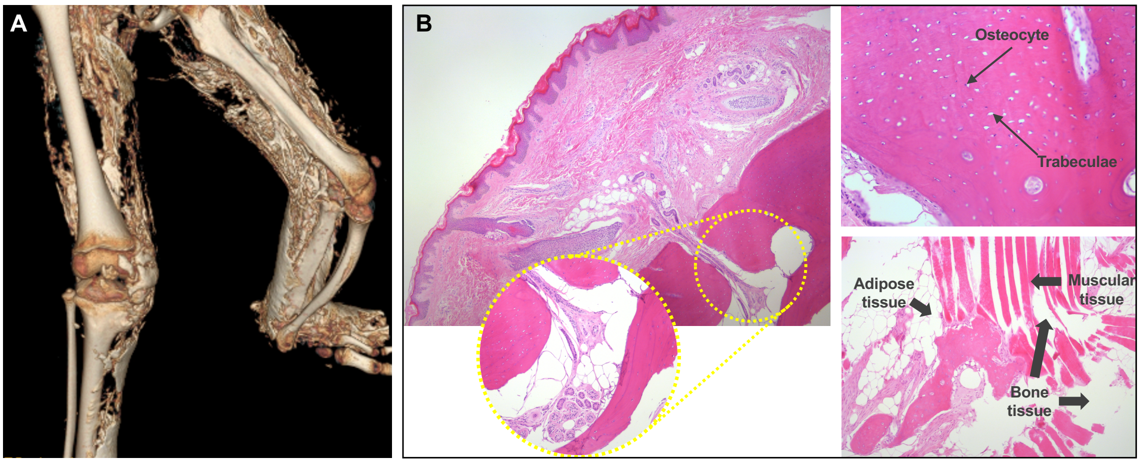

Fig. S2. Abdominal and scapular heterotopic ossifications collected from the patient.

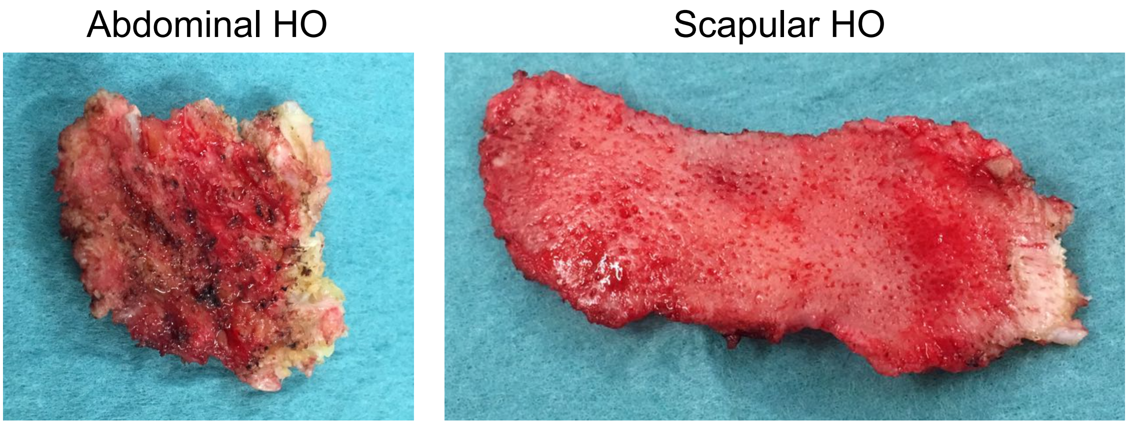

Fig. S3. Plot showing most differentially expressed genes with respect to control samples. The size of the bubbles is proportional to the log_2FC value and the color indicates over- or under-regulation with respect to control samples. Only genes with a $|log_2FC| > 2$ were included (and with >100 counts in any of the samples compared)

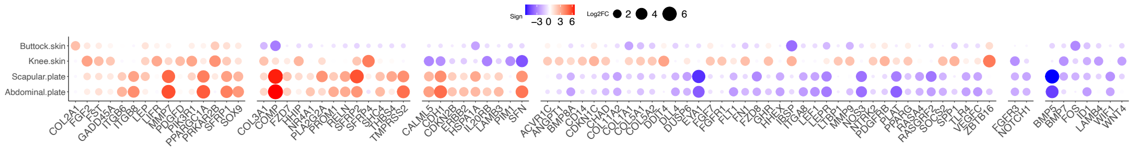

Fig. S4. Genes showing most different gene expression pattern between scapular and abdominal samples (A) and between knee and buttock samples (B) (log_2FC differences >1.5). Normalized expression values are represented in barplots, whereas log_2FC values between patient and control samples are shown in the bubble plot. expression values are represented in barplots, whereas log_2FC values between patient and control samples are shown in the bubble plot.

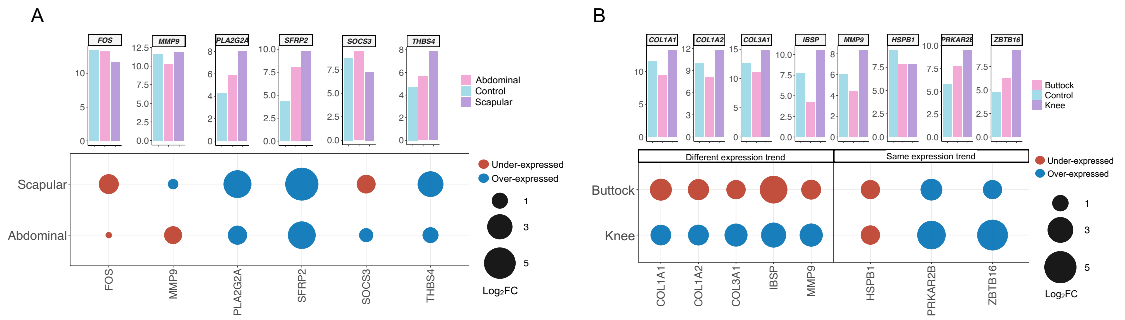

Fig. S5. Pathway analysis results from heterotopic ossification samples (A) and skin samples (B). Color gradient of the bars indicates the number of up or down-regulated genes included in the pathway gene-set. Only the top 30 most significant pathways are displayed.

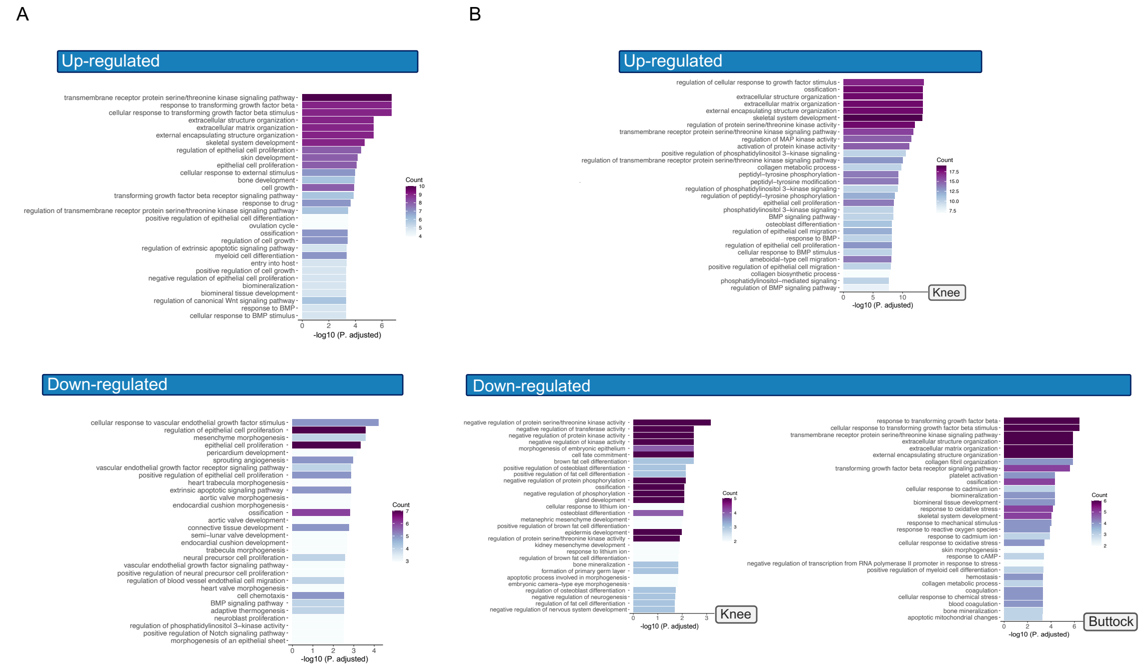

Fig. S6. (A) Pathways enrichment analysis from DEGs in blood samples showing the over-represented (A) and under-represented (B) significant terms (only the 30 top significant terms are displayed)

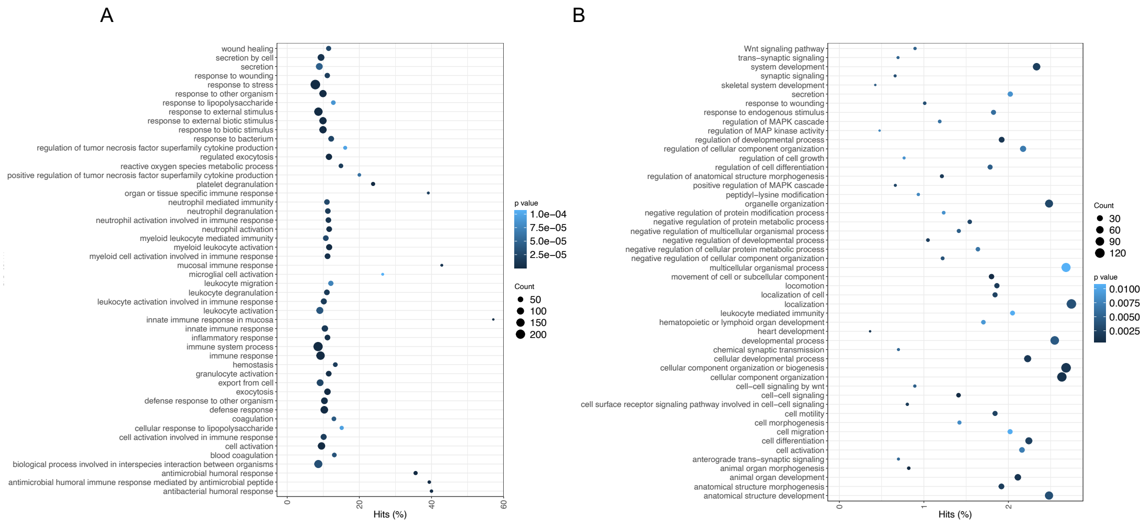

Fig. S7. (A) Target genes of the miRNAs most differentially expressed between blood samples from affected and non-affected twins. Only target genes with an aggregate rank value <5 are represented. Log_2FC values of miRNAs are also shown. (B) Network map showing the interaction between most differentially expressed miRNAs and the best predicted target genes. (C) Enrichment map of functional significant modules detected related to the best target candidates.

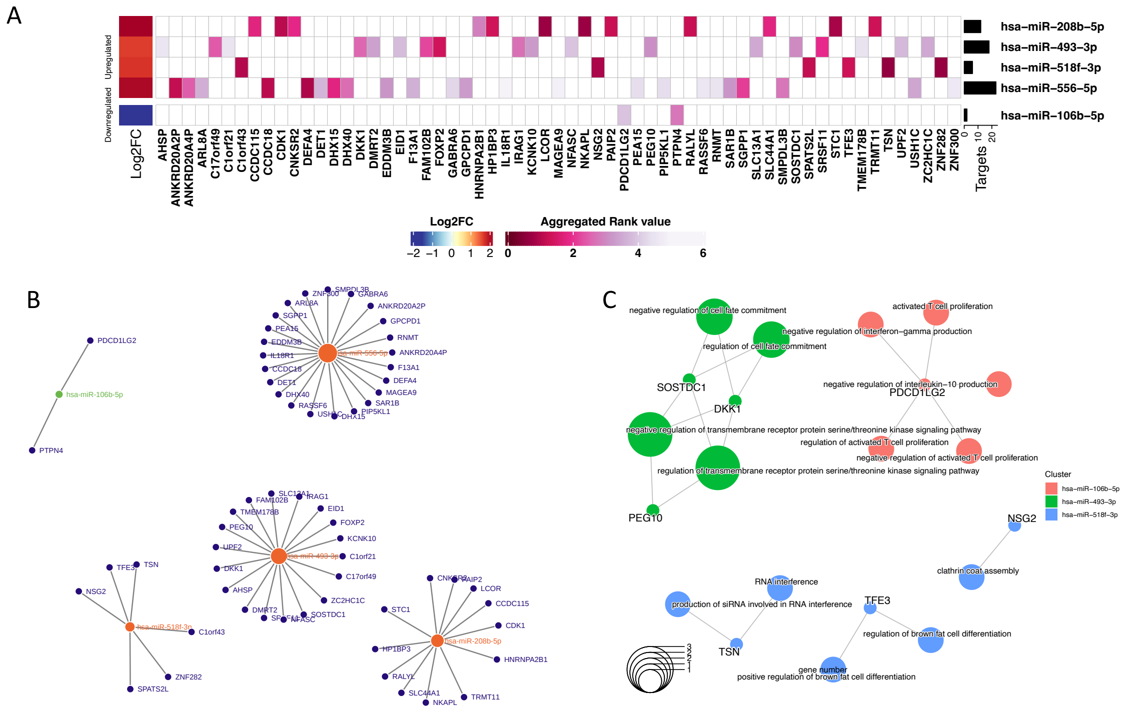

Supplement: Multimedia component 1 [file mmc1.pdf]
